# Genome-wide survey and analysis of microsatellites in nematodes, with a focus on the plant-parasitic species *Meloidogyne incognita*

**DOI:** 10.1186/1471-2164-11-598

**Published:** 2010-10-25

**Authors:** Philippe Castagnone-Sereno, Etienne GJ Danchin, Emeline Deleury, Thomas Guillemaud, Thibaut Malausa, Pierre Abad

**Affiliations:** 1 INRA UMR1301/UNSA/CNRS UMR6243, 400 Route des Chappes, BP167, Sophia Antipolis, France

## Abstract

**Background:**

Microsatellites are the most popular source of molecular markers for studying population genetic variation in eukaryotes. However, few data are currently available about their genomic distribution and abundance across the phylum Nematoda. The recent completion of the genomes of several nematode species, including *Meloidogyne incognita*, a major agricultural pest worldwide, now opens the way for a comparative survey and analysis of microsatellites in these organisms.

**Results:**

Using MsatFinder, the total numbers of 1-6 bp perfect microsatellites detected in the complete genomes of five nematode species (*Brugia malayi*, *Caenorhabditis elegans*, *M. hapla*, *M. incognita*, *Pristionchus pacificus*) ranged from 2,842 to 61,547, and covered from 0.09 to 1.20% of the nematode genomes. Under our search criteria, the most common repeat motifs for each length class varied according to the different nematode species considered, with no obvious relation to the AT-richness of their genomes. Overall, (AT)_*n*_, (AG)_*n *_and (CT)_*n *_were the three most frequent dinucleotide microsatellite motifs found in the five genomes considered. Except for two motifs in *P. pacificus*, all the most frequent trinucleotide motifs were AT-rich, with (AAT)_*n *_and (ATT)_*n *_being the only common to the five nematode species. A particular attention was paid to the microsatellite content of the plant-parasitic species *M. incognita*. In this species, a repertoire of 4,880 microsatellite loci was identified, from which 2,183 appeared suitable to design markers for population genetic studies. Interestingly, 1,094 microsatellites were identified in 801 predicted protein-coding regions, 99% of them being trinucleotides. When compared against the InterPro domain database, 497 of these CDS were successfully annotated, and further assigned to Gene Ontology terms.

**Conclusions:**

Contrasted patterns of microsatellite abundance and diversity were characterized in five nematode genomes, even in the case of two closely related *Meloidogyne *species. 2,245 di- to hexanucleotide loci were identified in the genome of *M. incognita*, providing adequate material for the future development of a wide range of microsatellite markers in this major plant parasite.

## Background

Microsatellites, also known as simple sequence repeats, are 1-6 base pair (bp) nucleotide motifs tandemly repeated in the genome of every eukaryotic organism analyzed so far. In contrast to non-repetitive DNA, microsatellite polymorphism is primarily due to variation in the number of repeated motifs rather than to substitutions. A very high mutation rate - from 10^-4 ^to 10^-3 ^mutation per microsatellite and per generation [1]- is usually associated with microsatellite loci, resulting in high heterozygosity and the presence of multiple alleles at a given locus [2]. These markers are co-dominant, abundant in non coding regions of the genome, are relatively easy to isolate, can be specifically amplified by PCR, and evolve according to mutation models that are well described [1,2]. Therefore, microsatellites have emerged as the most popular and versatile neutral markers for geneticists working on a wide range of topics including, among others, forensics, genome mapping, population structure, phylogeny, linkage and kinship relationships [3]. The most conventional procedure for the isolation of microsatellite markers, i.e., enrichment of genomic DNA for microsatellite motifs cloning, screening of the resulting library and sequencing of the positive clones [4], is challenging, time-consuming and costly. Such enrichment methods also generally use one or a few specific repeated motifs that are most often selected without prior knowledge of their abundance in the genome and may not produce suboptimal results. The recent availability of huge amount of sequence data for a wide range of organisms, together with new methodological developments of *in silico *mining of microsatellites, have tremendously increased the characterization of these markers [5], and will certainly catalyze the study of genomic distribution of microsatellites in eukaryotes.

The isolation of microsatellites as useable markers appears to be more difficult in some taxa than in others, and has proved to be difficult in many invertebrates, including nematodes [6,7]. Except for the model species *Caenorhabditis elegans*, whose genome has been sequenced in the past decade [8], no genome-wide survey of microsatellites is available for nematodes. In addition, there have been relatively few studies of microsatellites isolated by conventional molecular biology approaches in this phylum compared to other eukaryotes, e.g. insects or vertebrates. Such unpopularity of microsatellites as genetic markers in nematodes was attributed in part to the unusually high proportion of loci that fail to produce interpretable PCR patterns, possibly as the result of inter-locus flanking sequence similarity [9]. The root-knot nematode (RKN) *Meloidogyne incognita *is a serious plant parasite characterized by both its world-wide distribution and its very large host range [10], which raise questions about the origin, the processes of dispersal and the resulting genetic structure of the populations. In recent years, studies of genetic diversity have been carried out in this mitotic parthenogenetic organism using neutral molecular markers such as RAPD or AFLP, and revealed rather unexpected levels of clonal diversity among populations [11]. But surprisingly, like in other nematodes, microsatellites, which are usually regarded as among the most appropriate tools to study variation at the individual level, have been very poorly investigated in this taxon.

The recent completion of genome sequencing projects has provided new opportunities to evaluate and compare the distribution of microsatellites in nematodes. Besides the genome of *C. elegans*, additional whole-genome data are now available for nematodes with very different life styles, i.e. the necromenic species *Pristionchus pacificus *[12], the plant-parasitic species *M. incognita *and *M. hapla *[13,14], and the animal-parasitic species *Brugia malayi *[15]. Based on these genomic resources, we report here the first survey and comparative analysis of microsatellites in nematodes, which reveal variable patterns of microsatellite abundance and diversity in the genomes of these organisms. A more detailed focus on the genome of the RKN *M. incognita *allowed the characterization of 2,245 di- to hexanucleotide loci, providing the material basis for the future development of a wide range of microsatellite markers in this plant parasite of major agronomic importance.

## Results

### Relative abundance and diversity of microsatellites in nematode genomes

We examined the distribution of perfect 1-6 bp microsatellites using an optimized detection threshold of 12, 8, 5, 5, 5 and 5 repeats for mono-, di-, tri-, tetra-, penta- and hexa-nucleotide motifs, respectively. For each motif type, these are the minimum number of repeats required for a microsatellite to be reported, that have been optimized as default parameters of the software to eliminate repeats which might be observed by chance [16]. So the results described here apply to microsatellites meeting this criterion. Accordingly, the total numbers of microsatellites found in the five nematode genomes appeared highly variable, ranging from 2,842 to 61,547, and covered from 0.09 to 1.20% of the nematode genomes (Table 1). When considering density and coverage of the microsatellites (i.e., number and length per Mbp of analysed sequence, respectively) in the five genomes, three homogenous groups could be defined (Table 1). The first one comprised the two RKN species, *M. incognita *and *M. hapla*, which shared the lowest abundance of microsatellite loci. The second group combined *C. elegans *and *P. pacificus*, with microsatellite density and coverage values about two times higher than those of RKN. The largest values were exhibited by *B. malayi *(about five times those of *C. elegans*). The variability of microsatellite number found in nematode genomes was well explained by genome size for *M. incognita*, *M. hapla*, *C. elegans *and *P. pacificus *(*r*^2 ^= 0.95, *F*_1,2 _= 37.5, *p *= 0.03), but not when *B. malayi *was included (*r*^2 ^= 0.10, *F*_1,3 _= 0.34, *p *= 0.6) (Table 1).

**Table 1:** Global coverage and density of the microsatellite loci identified in five nematode genomes

	*M. incognita*	*M. hapla*	*C. elegans*	*P. pacificus*	*B. malayi*
Sequence analyzed (bp)	86,061,872	53,017,507	100,269,510	169,747,139	95,814,443
GC content (in %)	31.4	27.4	35.4	42.5	30.5
Number of microsatellite loci	4,880	2,842	11,382	20,694	61,547
Average density of loci (no./Mbp)	56.7	53.6	113.5	121.9	642.4
Total length of microsatellites (bp)	80,756	45,649	206,492	390,067	1,151,454
Coverage (length in bp/Mbp)	938.3	861.4	2,059.4	2,297.9	12,017.5
Genome content (in %)	0.09	0.09	0.21	0.23	1.20

Table 2 shows the relative abundance of the different microsatellite length classes (i.e., mono-, di-, tri- up to hexanucleotides). Overall, distributions significantly varied among species (Fisher exact test, *p *< 10^-4^). The *B. malayi *genome exhibited the highest density and coverage of microsatellites, except for 6-bp motifs that proved to be more frequent in *C. elegans*. Mononucleotide repeat motifs notably outnumbered all other length classes in the five nematode genomes, ranging from 48.7% of microsatellites in *M. hapla*, to 75.5% in *B. malayi*. Between species, there was a large variation in the number of mononucleotide repeats per Mb of genomic DNA, with a significant increase in *B. malayi*. After mononucleotides, trinucleotides were the next most abundant length class in nematode genomes, except for *B. malayi*. In this particular species, di- and trinucleotide motifs each represented about 10% of the total number of microsatellites. Quite surprisingly, dinucleotide motifs appeared underrepresented in the two RKN species, at 3.5 and 4% of the total number of microsatellites in *M. hapla *and *M. incognita*, respectively. Further, there was a drop in the frequency of longer motifs in the five nematode genomes. Exceptions were tetranucleotide motifs in *M. hapla*, and hexanucleotide motifs in *C. elegans*, which comprised 9.3 and 2.6% of the total number of microsatellites, respectively.

**Table 2:** Relative density and coverage of 1-6 bp microsatellites in five nematode genomes

Motif length (bp)		Nematode species				
		
		*M. incognita*	*M. hapla*	*C. elegans*	*P. pacificus*	*B. malayi*
1	No.	2,635	1,385	6,479	10,933	46,466
	No./Mbp	30.6	26.1	64.6	64.4	485.0
	%^a^	54.0	48.7	56.9	52.8	75.6
	Length (bp)	39,399	19,331	89,913	184,836	738,987
	Bp/Mbp	457.8	364.6	896.6	1088.9	7712.7
2	No.	196	99	1,982	3,971	7,058
	No./Mbp	2.3	1.9	19.8	23.4	73.7
	%	4.0	3.5	17.4	19.2	11.5
	Length (bp)	3,906	2,092	52,382	87,108	196,902
	Bp/Mbp	45.4	39.5	522.3	513.2	2055.0
3	No.	1,796	1,081	2,338	4,823	6,029
	No./Mbp	20.9	20.4	23.3	28.4	62.9
	%	36.8	38.0	20.5	23.3	9.8
	Length (bp)	28,629	16,902	39,291	88,401	137,445
	Bp/Mbp	328.5	318.8	391.8	520.8	1434.5
4	No.	213	265	246	709	1,661
	No./Mbp	2.5	5.0	2.5	4.2	17.3
	%	4.4	9.3	2.2	3.4	2.7
	Length (bp)	7,580	6,916	6,628	18,112	63,024
	Bp/Mbp	88.1	130.4	66.1	106.7	657.8
5	No.	34	10	38	163	224
	No./Mbp	0.4	0.2	0.4	1.0	2.3
	%	0.7	0.4	0.3	0.8	0.4
	Length (bp)	870	270	1,040	5,010	7,440
	Bp/Mbp	10.1	5.1	10.4	29.5	77.7
6	No.	6	2	300	95	109
	No./Mbp	0.1	0.1	3.0	0.6	1.1
	%	0.1	0.1	2.6	0.5	0.2
	Length (bp)	192	138	17,256	6,600	7,656
	Bp/Mbp	2.2	2.6	172.1	38.9	79.9

^a^% = no./total no. of microsatellites.

The most common repeat motifs for each length class varied with the different nematode species considered (Table 3). A_*n *_and T_*n *_repeats were the most frequent motifs in three genomes (*M. hapla*, *C. elegans *and *B. malayi*), while C_*n *_and G_*n *_repeats dominated in the two others (*M. incognita *and *P. pacificus*), with no obvious relation to the AT-richness of the genomes (*r*^*2 *^= 0.32, *F *= 1.39, *p *= 0.37). Overall, (AT)_*n *_, (AG)_*n *_and (CT)_*n *_were the three most frequent dinucleotide microsatellite motifs found in any of the five genomes considered, but with a variable relative rank in each species. The (AT)_*n *_motif was particularly dominant in *M. hapla *and in *B. malayi*. All together, these three motifs comprised from 69.4% (in *C. elegans*) to 94.7% (in *P. pacificus*) of the whole set of dinucleotide microsatellites in each genome. The frequency of tri- to hexanucleotide motifs was more variable, with the list of most frequent motifs becoming quite specific for each nematode species. Except for two motifs in *P. pacificus *(i.e., (CCT)_*n *_and (AGG)_*n*_), all the most frequent trinucleotide motifs were AT-rich, with (AAT)_*n *_and (ATT)_*n *_being the only common to the five nematode species. Tetra- to hexanucleotide repeats were much less common in all five genomes, except tetranucleotide motifs in *B. malayi*, and to a lesser extent hexanucleotide motifs in *C. elegans*, and none of these single motifs appeared to be shared by the five nematode species. Among these three length classes, only three of the most common motifs exhibited a GC content >50%, i.e. (AAGGG)_*n *_and (ACGGGG)_*n *_in *M. incognita*, and (ACCGGT)_*n *_in *C. elegans*. It is to be noted that no such GC-rich motifs occurred among the most frequent in *P. pacificus*, although this genome has the highest GC content. The (AACCCT)_*n *_telomeric-like hexanucleotide repeat [17] was found abundant only in the *B. malayi *genome.

**Table 3:** Relative frequency^a ^of the most frequent microsatellite motifs found in nematode genomes

Nematode species	Length of microsatellite motif (bp)					
		
	1	2	3	4	5	6
*M. incognita*	C (79.5)	AG (49.5)	AAC (52.2)	AAAT (54.0)	AAACG (70.6)	AAAAAT (33.3)
	A (20.5)	AT (27.0)	AAT (19.4)	AATT (24.9)	AAATT (8.8)	AACGGT (33.3)
	--	AC (23.5)	AAG (11.7)	AGGT (6.6)	AAGGG (5.9)	AAATTT (16.6)
	--	--	AGG (7.5)	AGGG (6.1)	AAAAG (2.9)	ACGGGG (16.6)
Total no./Mb^b^	30.6	2.3	20.9	2.5	0.4	0.1
*M. hapla*	A (75.0)	AT (66.7)	AAT (38.2)	AAAT (66.8)	AAAAT (50.0)	AAAATT (50.0)
	C (25.0)	AG (27.2)	AAC (37.5)	AATT (27.5)	CTTTT (20.0)	AAAGGG (50.0)
	--	AC (6.1)	AAG (12.1)	AACT (2.3)	AAATT (2.0)	--
	--	--	AGG (4.5)	AAAG (1.1)	AAACG (10.0)	--
Total no./Mb	26.1	1.9	20.4	5.0	0.2	0.1
*C. elegans*	A (72.8)	AG (36.7)	AAG (29.8)	ACCT (32.5)	AATTT (52.6)	AACCGT (57.0)
	C (27.2)	AT (32.6)	AAT (19.1)	AAAT (29.3)	AAACT (23.7)	AACGGT (8.3)
	--	AC (30.6)	ACT (17.0)	ACGG (12.2)	AACGT (7.9)	AAGTTT (6.7)
	--	CG (0.1)	ACG (12.4)	AAGT (9.3)	AAAAT (7.9)	ACCGGT (6.3)
Total no./Mb	64.6	19.8	23.3	2.5	0.4	3.0
*P. pacificus*	C (80.6)	AG (77.9)	AAT (26.7)	AAGT (29.1)	AACTT (52.8)	AACCGT (21.0)
	A (19.4)	AT (16.9)	AGG (20.5)	AATT (23.1)	AAAGT (11.7)	AAAAGT (18.9)
	--	AC (5.0)	AAC (17.9)	AACT (16.5)	AAACT (9.8)	AAACTT (10.5)
	--	CG (0.2)	AAG (17.4)	AAAT (9.9)	AAGGG (6.1)	AAGGGG (9.5)
Total no./Mb	64.4	23.4	28.4	4.2	1.0	0.6
*B. malayi*	A (99.6)	AT (44.7)	AAT (60.9)	AAGT (35.6)	AAAGT (36.6)	AACCGT (49.5)
	C (0.4)	AG (32.4)	ACT (17.2)	AACT (16.4)	AAGGT (13.4)	AACCCT (6.4)
	--	AC (22.8)	AAC (10.8)	AAAT (13.0)	AACTT (13.4)	AAAGGG (6.4)
	--	CG (0.1)	ACG (4.6)	AATT (9.8)	AAAAC (4.0)	ACGTTT (5.5)
Total no./Mb	485.0	73.7	62.9	17.3	2.3	1.1

^a^Most frequent motifs (maximum of 4) listed for each nematode species in each nucleotide class (frequency given as a percentage of the total number of microsatellites with a motif of the same length).

^b^Global density of microsatellites for each nucleotide class, including unlisted motifs (if any).

### Diversity of microsatellites in the genome of *Meloidogyne incognita*

**Figure 1:**
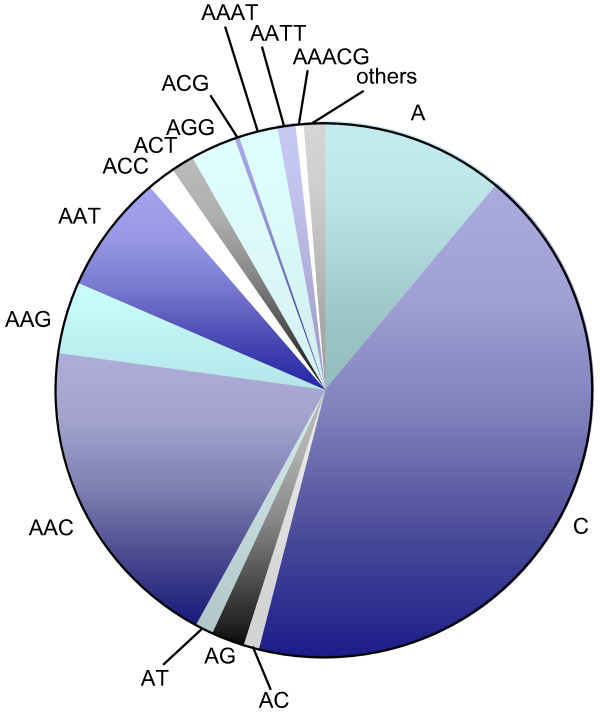
**Frequency of microsatellite classes in the genome of *Meloidogyne incognita***. Among the 58 classes identified, the 20 most frequent are shown in individual divisions. The remaining 38 microsatellites are considered in one single division defined as others. For the partial standardization method used to define microsatellite classes, see Methods.

We further focused our study specifically on the RKN *M. incognita*, because it is a plant-parasitic species of major economic importance for which molecular markers suitable for population genetics are not available. Using our search criteria, we could identify a repertoire of 4,880 microsatellite loci in the genome of this nematode, represented by 58 different motifs that covered 0.09% of the total genomic DNA analysed. Genomic location of microsatellites (i.e., their position on the genome contigs), motif types and motif iterations are given in the Additional file 1. The twenty most abundant microsatellite classes were C_*n*_, G_*n*_, (GTT)_*n*_, (AAC)_*n*_, T_*n*_, A_*n*_, (AAT)_*n*_, (ATT)_*n*_, (AAG)_*n*_, (CTT)_*n*_, (AGG)_*n*_, (CCT)_*n*_, (CT)_*n*_, (AAAT)_*n*_, (ATTT)_*n*_, (AT)_*n*_, (AATT)_*n*_, (ACC)_*n*_, (GGT)_*n *_and (ACT)_*n *_(Figure 1). Together, they comprised 95.6% of all microsatellites identified. Although they were the most frequent (Figure 1; Table 2), mononucleotide motifs were no longer considered in the following analyses, because they are not of interest as putative microsatellite markers. For the same reason, dinucleotide microsatellites were taken into account only when they had more than eight repeated units (and more than five units for the tri- to hexanucleotides).

Overall, 54 di- to hexanucleotide motifs were identified and contributed to the diversity of the microsatellite repertoire in the *M. incognita *genome (Table 4). Small number (≤10) of tandem repeats of the microsatellite motifs predominated with more than 97% of all motifs belonging to this length group. A declining trend in abundance according to the number of repetitions occurred for all motifs, except for the tetranucleotide (AGGT)_n_. Interestingly, this motif also comprised the most iterated microsatellite locus, spanning 124 repetitions. Microsatellites consisting of trinucleotide repeats were by far the most abundant, representing 80% of the di- to hexanucleotides found in the nematode genome. They were followed by tetra- and dinucleotides, at a relative frequency of 9.5 and 8.7%, respectively, while penta- and hexanucleotides appeared quite rare (1.7 and 0.3%, respectively). Five different dinucleotide motifs were identified, that were comparable in terms of frequency (from 1 to 2.7%). Conversely, the distribution was extremely variable among the 15 trinucleotide motifs characterised, with frequencies ranging from 0.3 to 20.5%. The most frequent motifs were the AT-rich (GTT)_*n *_and (AAC)_*n*_, which collectively represented more than 41% of the total number of di- to hexanucleotides identified in the *M. incognita *genome. In contrast, the only motif composed solely of C and/or G, (CGG)_*n*_, was very poorly represented (0.09%). Overall, a total of 99 microsatellites (i.e., 66 di-, 13 tri- and 20 tetranucleotides) exhibited a large number of repeated motifs (≥10).

**Table 4:** The di- to hexanucleotide microsatellites found in the genome of *Meloidogyne incognita*

Motif length	Motif	Iteration				Total no. (%)	Longest locus
				
		≤10^a^	11-20	21-30	>30		
2	AC	37	7	1	1	46 (2.05)	(AC)_*51*_
	AG	82	14	1	-	97 (4.32)	2× (AG)_*21*_
	AT	44	7	1	1	53 (2.36)	(AT)_*47*_
3	AAC	934	1	-	-	935 (41.65)	(AAC)_*11*_
	AAG	211	-	-	-	211 (9.40)	2× (AAG)_*10*_
	AAT	346	3	-	-	349 (15.55)	(AAT)_*14*_
	ACC	83	-	-	-	83 (3.70)	3x (ACC)_*9*_
	ACT	67	-	-	-	67 (2.98)	2× (ACT)_*10*_
	AGG	134	1	-	-	135 (6.01)	(AGG)_*11*_
	ACG	14	-	-	-	14 (0.62)	14x (ACG)_*5*_
	CGG	2	-	-	-	2 (0.09)	2× (CGG)_*5*_
4	AAAC	6	-	-	-	6 (0.27)	(AAAC)_*7*_
	AAAT	110	3	1	1	115 (5.12)	(AAAT)_*41*_
	AAAG	1	-	-	-	1 (0.04)	(AAAG)_*6*_
	AATT	53	-	-	-	53 (2.36)	(AATT)_*10*_
	AAGG	3	-	-	-	3 (0.13)	(AAGG)_*10*_
	AGGG	13	-	-	-	13 (0.58)	2× (AGGG)_*10*_
	AGGT	4	1	-	9	14 (0.62)	(AGGT)_*124*_
	AGTT	1	-	-	-	1 (0.04)	(AGTT)_*5*_
	ACGT	1	-	-	-	1 (0.04)	(ACGT)_*5*_
	AACT	1	-	-	-	1 (0.04)	(AACT)_*7*_
	AACG	3	-	-	-	3 (0.13)	3x (AACG)_*5*_
	GGGT	1	-	-	-	1 (0.04)	(GGGT)_*5*_
	ACTT	1	-	-	-	1 (0.04)	(ACTT)_*5*_
5	AAATT	3	-	-	-	3 (0.13)	(AAATT)_*7*_
	AAACG	24	-	-	-	24 (1.07)	(AAACG)_*6*_
	AAAAG	1	-	-	-	1 (0.04)	(AAAAG)_*5*_
	CCTTT	1	-	-	-	1 (0.04)	(CCTTT)_*5*_
	AAGGG	2	-	-	-	2 (0.09)	(AAGGG)_*5*_
	AGGGG	1	-	-	-	1 (0.04)	(AGGGG)_*5*_
	AGTTT	1	-	-	-	1 (0.04)	(AGTTT)_*5*_
	CGGTT	1	-	-	-	1 (0.04)	(CGGTT)_*5*_
6	AAAAAT	2	-	-	-	2 (0.09)	2× (AAAAAT)_*5*_
	AAATTT	1	-	-	-	1 (0.04)	(AAATTT)_*5*_
	ACGGGG	1	-	-	-	1 (0.04)	(ACGGGG)_*7*_
	AACGGT	2	-	-	-	2 (0.09)	2× (AACGGT)_*5*_
	Total	2192	37	4	12	2245	

^a^For dinucleotides, n ≥ 8; for tri- to hexanucleotides, n ≥ 5.

### Distribution of microsatellites in the coding regions of the genome of *Meloidogyne incognita *and Gene Ontology annotation

**Figure 2:**
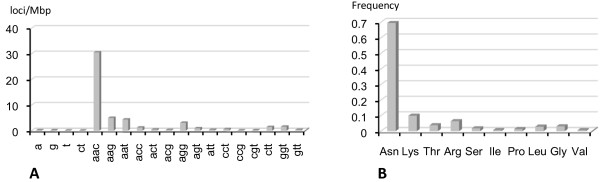
**Microsatellites detected in the coding sequences of *Meloidogyne incognita***. (A) Density of microsatellites (expressed as the number of loci/Mb coding sequence). (B) Frequency of the amino acid repeats encoded by the trinucleotide microsatellites.

Among the 20,365 predicted protein-coding loci (CDS) searched, a total of 1,094 microsatellites were identified in 801 CDS, including 181 CDS containing at least two microsatellites (Additional file 2). They represent 22.4% of the total number of microsatellite loci found in the whole genome of this nematode. Overall, the distribution density of microsatellites is 53.7 per Mb of coding sequence. The occurrence of the repeat motifs found is shown in Figure 2A. Only mono-, di- and trinucleotide motifs were observed, this last category representing more than 99% of the total set of microsatellites. With 64.2% of repeat motifs, (AAC)_n _turned out to be the most frequent microsatellite, followed by (AAG)_n _(9.9%), (AAT)_n _(8.6%) and (AGG)_n _(6.0%). Among trinucleotide motifs, GC-rich microsatellites represented 13.6% only of the total number of loci. We further investigated the frequency of the amino-acid repeats (AAR) encoded by the tri-nucleotide repeats in the CDS (Figure 2B). The ten different amino acids that composed the AAR identified were, from the most to the least frequent, Asparagine (Asn), Lysine (Lys), Threonine (Thr), Arginine (Arg), Serine (Ser), Isoleucine (Ile), Proline (Pro), Leucine (Leu), Glycine (Gly) and Valine (Val). The longest AAR encoded by trinucleotide motifs was observed for Asn with 14 repeats.

**Figure 3:**
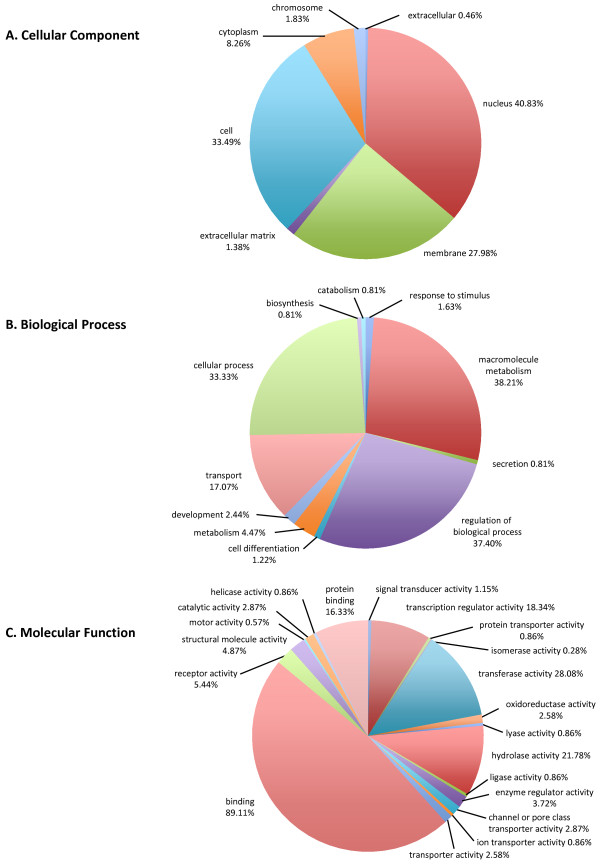
**Gene Ontology (GO) assignment of the *Meloidogyne incognita *predicted proteins encoded by CDS containing microsatellite motif(s)**. In each of the three GO categories, the percentages of annotated sequences do not add up to 100% because some predicted proteins have more than one GO category assigned to them.

When scanned for InterPro domains, 497 out of the 801 CDS (~62%) containing at least one microsatellite motif were found to harbour at least one known domain (Additional file 3), and were further assigned a corresponding Gene Ontology (GO) term. Overall, Cellular Component, Molecular Function and Biological Process GO terms could be assigned to 218, 349 and 246 CDS, respectively. For each GO category, the relative distribution of GO terms is represented in Figure 3. With regard to the cellular component category, 40.8% of the CDS were assigned a nucleus GO term, followed by the cell (33.5%) and the membrane (28%) categories. Conversely, the extracellular (0.5%) and extracellular matrix (1.4%) GO terms were poorly represented. Macromolecule metabolism (38.2%) was the most frequent GO term for CDS in the biological process category, just followed by regulation of biological process (37.4%) and cellular process (33.3%). Notably, only 0.8% and 1.6% of the annotated CDS were assigned a secretion and response to stimulus GO term, respectively. When the molecular function category was considered, binding GO term was over-represented (89.1%), followed by transferase (28.1%) and hydrolase (21.8%) activities.

## Discussion

### Diversity of microsatellite distribution in nematode genomes

In this study, we used Msatfinder [16] to scan the recently assembled *M. incognita*, *M. hapla*, *B. malayi *and *P. pacificus *genomes for perfect microsatellites of 1-6 bp long. To validate our results, we performed a similar analysis of the *C. elegans *genome using the same bioinformatics tool and search parameters. The coverage of microsatellite loci in *C. elegans *has previously been estimated at 2139 bp/per Mbp [18], and we were encouraged in that our results are consistent with those previously reported for this nematode (~2059 bp/Mbp; Table 1). Indeed, such a consistency between two independent studies may be considered as a strong indication of the robustness of the global analysis.

It has long been considered that the structure and organization of the *C. elegans *genome would serve as accurate model for most nematodes [19]. Previous comparative studies of model eukaryotes have shown that *C. elegans *has the lowest frequency and coverage of microsatellites in its genome, even less than *Saccharomyces cerevisiae *and other fungi [18,20,21]. Our own analysis reveals a huge variation among the five nematode genomes investigated in terms of both coverage and density of microsatellites, that is independent from the size of the genomes considered. While *P. pacificus *has similar microsatellite density and coverage than *C. elegans*, the two RKN species, *M. hapla *and *M. incognita*, both exhibit a twofold lower abundance of microsatellite loci. Conversely, the animal parasite *B. malayi *contains five times more microsatellites/Mbp than *C. elegans*. Phylogenetic relationships within the Nematoda phylum have been considered to tentatively explain such variability. Based on small subunit ribosomal DNA sequences, the phylogeny of nematodes identifies five major clades in the phylum [22]. In this framework, *C. elegans *and *P. pristionchus *belong to Clade V, *M. incognita *and *M. hapla *belong to Clade IV, and *B. malayi *belong to Clade III, respectively. Although a loose correlation may be seen here, additional data from many other nematode species from all five clades are required before considering any hypothetical relationship between the clade of origin and the density of microsatellites for a given species. Clearly, these data provide evidence of variable patterns of microsatellite distribution in nematode genomes, indicating that the particular contribution of these shorts tandem repeats to the genome of *C. elegans *may not be the rule for other nematodes.

Without taking into account mononucleotide repeats, it is generally reported that dinucleotides are the most common microsatellites in many organisms [23,24]. Here, dinucleotides appeared significantly underrepresented in both RKN species, with as few as 3.5 and 4.0% of the total number of microsatellites in *M. hapla *and *M. incognita*, respectively. Conversely, trinucleotides were overrepresented in both the two latter species compared to *C. elegans*, *P. pacificus *and above all *B. malayi*. Such a distribution bias in favour of trinucleotides has been reported for some other eukaryotes, e.g., the fungus *Neurospora crassa *[25] or the insect *Tribolium castaneum *[26]. Also, a recent data-mining analysis of ESTs from phytoparasitic nematodes, including some RKN species, showed that trinucleotide repeats were the most abundant microsatellites in coding ESTs [27].

Data-mining of 26 completed genomes showed that microsatellites with low GC content are predominant in most eukaryotic genomes [5]. This trend also emerged from our survey, with the majority of the most frequent 1-6 bp microsatellite motifs from nematodes being AT-rich. However, one notable exception was constituted by polyG/polyC mononucleotide repeats in *M. incognita *and *P. pacificus*. Conversely, none of the most frequent di- to hexanucleotide repeats contains exclusively Cs or Gs. Among nematode dinucleotides, (AT)_n _motifs seem to be predominant compared with other motifs, while (CG)_n _were extremely rare, and even absent in the two RKN species. (CT)_n _motifs, which are the most abundant dinucleotides in insects [28] and other invertebrates [20], were also frequently detected in nematode genomes. In the same way, trinucleotides were dominated by AT-rich motifs, with (ATT)_n _and (AAT)_n _being always present in the four most common motifs in the five species investigated. The only exceptions were (CCT)_n _and (AGG)_n_, which were frequently detected in the *P. pacificus *genome. AT-richness was further exhibited by tetra- to hexanucleotides, with some remarkable exceptions, i.e., (AAGGG)_n _and (ACGGGG)_n _in *M. incognita*, or (ACCGGT) _n _in *C. elegans*. Overall, the diversity of microsatellite motifs gave each of the five nematode species a unique pattern of repeat distribution, even in the case of the two related RKN species tested here, suggesting that they can be efficient at differentiating those species.

### Microsatellites in the protein-coding sequences of *Meloidogyne incognita*

A total of 1,094 perfect microsatellites have been identified from the whole *M. incognita *CDS dataset, i.e., 3.9% of protein-coding sequences possess such repeats. The vast majority (>99%) of them are trinucleotide repeats. This result confirms a recent report indicating that trinucleotide repeats were the most abundant microsatellites in coding ESTs from 16 species of plant-parasitic nematodes belonging to seven genera, including *Meloidogyne *[27]. Moreover, in agreement with these authors, we found that (AAC)_n _repeats were the most abundant in *M. incognita *CDS, while (ATG)_n _or (TTA)_n_, that could act as start or stop codon, respectively, were not detected. The proportion of trinucleotide repeats in *M. incognita *CDS exponentially decreased as the number of repeats increased, with 14 repeats being a critical threshold. It is hypothesized that longer repeats are eliminated by selection acting on the nematode genome, since long amino acid repeats may have detrimental effects on protein functions [29,30]. In addition, our data showed that the hydrophilic amino acid Asn, and to a lesser extent Lys and Arg, are over-represented among the runs of amino acids in *M. incognita *proteins, which is consistent with the observation that stretches of hydrophilic amino acid are more tolerated in proteins [29,30].

A broad range of functions have been ascribed to amino acid repeats in proteins, including roles in intracellular protein-protein interactions, binding to host-cell receptors and polymerisation of their associated, non-repeated domains [31]. In parasitic eukaryotes such as *Trypanosoma brucei *or *Plasmodium falciparum*, protein repeats are often implicated in antigenic recognition and evasion of the host immune response to infection [32]. Mining the *M. incognita *predicted proteome revealed a set of 801 proteins containing stretches of amino acid repeats, of which 497 had at least one InterPro domain assigned, thus illustrating the importance of these peculiar structures in parasitic nematodes too. In addition, GO annotation revealed that binding was the molecular function preferentially assigned to these proteins. Based on the hypothesis that amino acid repeats can mediate interactions between the parasite and its host, and thus could potentially have a role in pathogenicity [33,34], a similar role for (some of) these proteins identified in *M. incognita *may be proposed. From this point of view, our analysis generated a large set of candidate proteins for further functional analysis in relation to pathogenicity in plant nematodes.

### Microsatellites as genetic markers in *Meloidogyne incognita*

Due to the relatively small size of nematode genomes, and the high impact of many parasitic species on animal/human health or agricultural production, one of the expected outputs of nematode genome projects is the development of reliable and informative molecular markers usable as genotyping tools in population studies. Among the nematode species included in this work, and except for the model organism *C. elegans*, microsatellite markers have been very poorly developed, and their practical application has been limited. Thus, a significant output of the present survey has been the identification of a wealth of microsatellite motifs in each of the nematode genomes analyzed. In the case of plant-parasitic nematodes, population genetics studies based on the use on polymorphic microsatellite markers are very scarce. They mainly concern the cyst nematodes *Globodera pallida *[35-37] and *Heterodera schachtii *[38], as well as the pinewood nematode *Bursaphelenchus xylophilus *[39], the reniform nematode *Rotylenchulus reniformis *[40] and the dagger nematode *Xiphinema index *[41]. Conversely, because of the lack of microsatellite markers available, no such study has been developed on *M. incognita*, although this species has been considered as 'the world's most damaging plant pathogen' [10]. Indeed, only one microsatellite locus had been characterized so far in RKN, i.e. in *M. artiellia*, a species assumed to reproduce both by amphimixy and facultative meiotic parthenogenesis [42]. The present study, with a total of 2,245 di- to hexanucleotide loci identified in the genome of *M. incognita*, now opens new perspectives for the development of a wide range of microsatellite markers in this nematode. Moreover, it provides useful information about possible physical linkage between microsatellite loci and identifies markers located in coding regions that may not be considered as neutral.

From a more general point of view, the microsatellites identified in this study may be useful for linkage analysis in the context of genetic mapping. Although not available for the parthenogenetic RKN *M. incognita*, genetic maps have been extensively developed in many nematode species, including *M. hapla *[14], *C. elegans *[43] and *P. pacificus *[44] and should benefit from the input of large batches of new markers. Additionaly, in the two latter species, location of microsatellites on sex chromosomes may be of further help for kinship analysis. To our knowledge, only one study reported the genome-wide chromosomal location of microsatellites in *C. elegans*, with no significant distorsion in frequency and distribution on the X chromosome compared to autosomes [20]. Conversely, no such information can be provided for RKNs, due to the lack of sex chromosomes in these species, where sex determinism is under environmental epigenetic control [45,46]. However, microsatellite loci could be located on the 2,995 contigs resulting from the genome assembly of the nematode [13], and this information has been obtained for the 4,880 microsatellite loci presently identified.

Because of its negligible cost, *in silico *mining of microsatellites makes this approach more comfortable than bench screening of genomic libraries. Although further experimental work is needed to setup PCR protocols and select the polymorphic loci that will become usable as genetic markers, recent genetic mapping or DNA fingerprinting studies demonstrated the success of this strategy [5]. For example, in the insect *T. castaneum*, 509 new polymorphic markers were experimentally validated from 12,160 loci identified by a bioinformatics analysis [47]. Of the 2,245 di- to hexanucleotide microsatellite sequences discovered in *M. incognita*, 2,183 had sufficient flanking sequences to allow the design of primer pairs. These data, including primer sequences, may become useful for developing variable markers in *M. incognita*.

## Conclusions

This analysis of microsatellites in completely sequenced nematode genomes provides a snapshot of the differential coverage and density of 1-6 bp repeats among the five species investigated. In particular, the two RKN species, *M. hapla *and *M. incognita*, both exhibit a two times lower abundance of microsatellite loci than *C. elegans*, which was previously considered as containing the lowest frequency of microsatellites among (model) eukaryotes. Quite surprisingly, dinucleotide motifs appeared underepresented in the two RKN species compared to the other nematode genomes, while trinucleotides were equally overrepresented in the two latter species compared to *C. elegans*, *P. pacificus *and *B. malayi*. The focus on *M. incognita *led to the identification of 4,880 microsatellites, 2,183 of them being *a priori *suitable to design markers for population genetics. Interestingly, 22.4% of the detected microsatellites were located in coding regions, almost all being short (<14 repetitions) trinucleotide motifs. This result suggests that microsatellites may affect evolution of proteins structure and function in this species.

The nucleotide sequences of the microsatellite loci obtained in this work, together with their flanking regions and amplification primers, are available for the five nematode species, upon request to the authors. Undoubtedly, this information may become useful for the development of large sets of markers that should in turn allow linkage mapping studies and facilitate population genetic research on nematodes.

## Methods

### Sequence data

The *M. incognita *and *M. hapla *genome assemblies [13,14] were downloaded directly from the sequencing project websites at http://www.inra.fr/meloidogyne_incognita and http://www.hapla.org. respectively. The genome sequence of *C. elegans*, *B. malayi *and *P. pacificus *were downloaded from WormBase (release WS210) at http://www.wormbase.org/. The total number of bp searched and (G + C) content for each of the three genomes are indicated in Table 1. For *M. incognita*, the whole set of 20,365 protein-coding sequences (including splice variants) predicted from the whole-genome sequence [13] was included in the analysis.

### Sequence analyses

Genomic sequences were scanned for microsatellite content using the program Msatfinder v2.0.9 [16] downloaded at http://www.genomics.ceh.ac.uk/msatfinder/. Msatfinder is a Perl script designed to allow the identification and characterisation of microsatellites in a comparative genomic context. The Regex search engine, which implements fast regular expressions to search once through the sequence, was used in our analyses. Detection criteria were constrained to perfect repeat motifs of 1-6 bp and a minimum repeat number of 12, 8, 5, 5, 5 and 5, for mono-, di-, tri-, tetra-, penta- and hexa-nucleotide microsatellites, respectively. Primer pairs for the identified microsatellite loci were designed using the Primer3 software [48] implemented in Msatfinder using default parameters.

Partial standardization of the microsatellite motifs was used for categorizing and comparing microsatellites, by considering overlapping components occurring in one DNA strand only, i.e. without including the sequence complement [49]. For example, the AAC class contains the (AAC)_n_, (ACA)_n _and (CAA)_n _microsatellites. In the present study, a total of 4, 6, 20, 60, 204 and 670 theoretical classes were considered for mono, di, tri, tetra, penta and hexanucleotides, respectively. To allow direct comparisons regardless of the size of the genomes analysed, density (number of loci) and coverage (number of bp) of the microsatellites were calculated for 1 Mbp of the corresponding genomic sequence.

The complete set of predicted protein-coding sequences resulting from the *M. incognita *genome project [13] was scanned for InterPro domain content [50]. Based on the domain annotations, Gene Ontology (GO) terms were assigned using the annotation tools available on the GO consortium website http://www.geneontology.org. GO annotations were further formatted for input into the GOSlim program and the output was parsed to count the occurrence of each GO category.

## Authors' contributions

PCS conceived and coordinated the study, participated in the data analysis and drafted the manuscript. EGJD performed the Msatfinder analyses and participated in the writing of the manuscript. ED carried out the Gene Ontology annotation. TG and TM both participated in the data analysis and in the writing of the manuscript. PA participated in the design of the study and assisted in the manuscript preparation. All authors read and approved the final manuscript.

## Supplementary Material

Additional file 1 **Table S1: The 4,880 microsatellite loci identified in the genome of *Meloidogyne incognita***. The 'primers' row indicates the microsatellites for which amplification primers could be designed (Y) or not (N) using the Primer3 software [48]. Primers are available upon request to the authors. Click here for file

Additional file 2 **Table S2: The 1,094 microsatellite loci identified in the protein-coding sequences of *Meloidogyne incognita***. The 'primers' row indicates the microsatellites for which amplification primers could be designed (Y) or not (N) using the Primer3 software [48]. Primers are available upon request to the authors. Click here for file

Additional file 3 **Table S3: InterPro annotation of the *Meloidogyne incognita *protein-coding sequences containing microsatellite motif(s)**. Click here for file
